# Diné Navajo Resilience to the COVID-19 pandemic

**DOI:** 10.1371/journal.pone.0272089

**Published:** 2022-08-04

**Authors:** Wilfred F. Denetclaw, Zara K. Otto, Samantha Christie, Estrella Allen, Maria Cruz, Kassandra A. Potter, Kala M. Mehta

**Affiliations:** 1 Department of Biology, San Francisco State University, San Francisco, California, United States of America; 2 Department of Public Health, San Francisco State University, San Francisco, California, United States of America; 3 Department of Epidemiology and Biostatistics, University of California San Francisco, San Francisco, California, United States of America; George Mason University, UNITED STATES

## Abstract

**Objective:**

To date, there are no studies of COVID-19 cases and deaths in the Navajo Nation, US. The primary objective of this manuscript is to understand whether counties with a higher proportion of Navajo (Diné) population also had higher cases and deaths of COVID-19 and whether these dropped with vaccination.

**Method:**

We undertook a cross-sectional analysis of county level data from March 16, 2020—May 11, 2021. Data were obtained from public repositories and the US Census for the Navajo Nation, including northeastern Arizona, southeastern Utah, and northwestern New Mexico. The primary outcome measure is the number of individuals with confirmed cases or deaths of COVID-19. A secondary outcome was COVID-19 vaccinations.

**Results:**

The 11 counties in Navajo Nation have a wide variation in the percent Navajo population, the resources available (ICU beds and occupancy), and COVID-19 outcomes. Overall, there was a substantial increase in the number of cases from March 16 –July 16, 2020 (the height of the pandemic) with a doubling time of 10.12 days on Navajo Nation. The percent Navajo population was a strong predictor of COVID-19 cases and deaths per million population. COVID-19 vaccinations were inversely associated with COVID-19 cases and deaths in these counties.

**Conclusions:**

The COVID-19 pandemic on the Navajo Nation is a story of resilience. Navajo Nation was one of the hardest hit areas of the United States, with peak cases and deaths due to COVID-19. With an aggressive vaccination effort, these cases and deaths were strikingly curtailed, showing the resilience of the Navajo (Diné) people.

## Introduction

In Navajo culture, introduction is not by a first and last name, rather introductions include clan relationships, birthplace, and residence. In the Navajo Nation, Navajo traditions and culture have a strong presence [1,2]. On March 11, 2020 the SARS-CoV2 coronavirus made an unceremonious entrance on the Navajo Nation in Chilchinbeto, AZ. Shortly thereafter, COVID-19 was named Dikos Ntsaaígíí- (“big cough”) in the Navajo language [3]. In a span of 2 months, Navajo Nation surpassed all US states with the highest per capita infection rate. According to the Navajo Epidemiology Center, as of May 11, 2021, the Navajo Nation reached 30,578 positive cases of SARS-CoV2 infections and 1,283 confirmed deaths [4].

The rapid spread and high numbers of SARS-CoV2 infection among the Navajomay be borne out of a failure of the US government trust responsibilities to Indian tribes [5]. In exchange for tribal lands, resources, and a peaceful co-existence, the US government promised tribes federal aid, infrastructure support, and protection through approximately 371 signed treaties [6].

However, after two centuries of federal government short falls in treaty promises to Indian tribes, a sustained low-level funding of tribes and neglect of basic infrastructure (physical, social, and economic) has today produced grievous inadequate living conditions and high rates of poverty on federal Indian reservations, including the Navajo Nation [7]. The rapid early spread of the pandemic on the Navajo Nation brings out into the open the effects of historic neglect and gross inequities faced by residents of the Navajo Nation and other Indian tribes.

Inequitable access to social determinants of health put the Navajo people at high risk for infectious diseases. A higher risk of COVID-19 infections may have been conferred by more public-facing occupations, similar to African American and Latinx or Latine individuals [8,9], who had higher rates due to their roles as essential workers, for example. Communities where multigenerational homes are common with family members providing care for both children and elders—were also at higher risk. Physical isolation is difficult in these contexts. Simultaneously, adult household members may commute long distances together to collect provisions from the closest store [10]. Social distancing may have been difficult outside the home as there are very few grocery stores, laundry mats, gas stations, and convenience stores, all used by the same individuals [11]. In addition, risk behaviors such as tobacco smoking and alcohol consumption [12], and pre-existing health conditions and comorbidities such as obesity, heart disease, diabetes [13], liver and kidney disease [14,15] challenge Indian tribes people to address COVID-19 disease on their reservation in addition to other health concerns. Furthermore, health care facilities on the Navajo Nation that may have been appropriate in 1960 when many were established but they may not have enough beds or workers to mount a response to a global pandemic [16–18]. Lastly, approximately one-third of homes on the Navajo Nation lack piped water and/or electricity, both essential infrastructure needs to combat COVID-19. With this backdrop, our team, led by a Navajo biologist, sought to document COVID-19 cases, deaths, and vaccinations in the Navajo Nation from March 16, 2020, to May 11, 2021.

## Methods

This study was reviewed by the Institutional Review Board (IRB) at University of California, San Francisco.

### Setting and population

Navajo Nation is an American Indian territory covering 27,000 square miles, including northeastern Arizona, southeastern Utah, and northwestern New Mexico. Further, it is comprised of five regional agencies with 110 chapter communities and a centralized three-branch government [19]. We focused on the eleven counties that intersect with Navajo Nation: Coconino, Navajo, Apache (Arizona); McKinley, San Juan, Cibola, Sandoval, Bernalillo, Rio Arriba and Socorro (New Mexico), and San Juan (Utah).

### Measures

Primary measures in this study were confirmed cases and deaths of COVID-19. Cases were determined by RT-PCR nasopharyngeal swab tests. Confirmed COVID-19 deaths were reported by the Indian Health Service and county-level departments of public health. COVID-19 cases and deaths per million population were derived from the New York Times GitHub [20] and the percent of Navajo population, per county, were derived from a report by the Navajo Epidemiologic Center [19]. Resources available, including Intensive Care Unit (ICU) beds and occupancy were derived from Kaiser Health News [21]. From these data we calculated death-to-case ratio (number of deaths/number of cases per county) and doubling time (number of days passed*ln(2))/(ln(end day cases/ beginning day total cases).

### Statistical analyses

Confirmed cases of COVID-19 overall for Navajo Nation were plotted using an arithmetic and a logarithmic scale, from March 16, 2020 to May 11, 2021. For the eleven counties within the Navajo Nation, we examined the total ICU beds and the estimated ICU bed occupancy.

We also examined the relationship between percent of the population self-identified as Navajo as the primary predictor, and the three COVID-19 metrics as the primary outcomes. We utilized linear regression with county as the unit. Further, to explore whether healthcare access affects COVID-19 outcomes, we examined the addition of occupancy of ICU beds per county to each model. All analyses were performed using STATA version 16.1.

## Results

**Fig 1:**
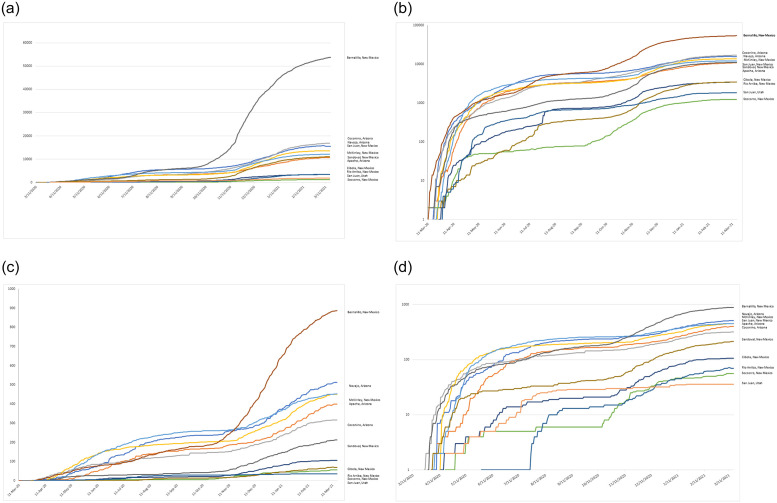
Number of confirmed COVID-19 cases from March 16, 2020, to May 11, 2021, by county, on the Navajo Nation plotted on an arithmetic (A & C) and logarithmic (B & D) scale.

There was a wide variation among the counties in the percent Navajo population, the resources available (ICU beds and occupancy) and COVID-19 cases, deaths, death-to-case ratios, and doubling time (Table 1). Confirmed cases of COVID-19 in counties in the Navajo Nation were plotted using arithmetic and logarithmic plots to reveal substantial increases in COVID-19 cases and deaths, followed by a tapering off with availability of vaccine, with a doubling time of 10.12 days early in the pandemic, elongating to 32.30 days by March 2021(Table 1). We show a steady increase in COVID-19 cases from March 2020 to March 2021 on both a linear and logarithmic scale. COVID-19 case trends and COVID-19 death trends are parallel (Fig 1).

**Table 1:** Counties from three US States in the Navajo Nation with percent Navajo population and COVID-19 case attributes.

	Total Population	Total COVID-19 Cases^1^	Total COVID-19 Deaths^1^	Total ICU Beds per county^2^	Estimated ICU beds needed^2^	Death-to-case ratio	Doubling time (days)	Percent of population self-identified Navajo^3^	Population self-identified Navajo
**Arizona**									
Apache	71,518	11,360	429	0	0.6	0.037	35.08	61%	43,626
Coconino	134, 421	17,863	330	41	1.4	0.019	32.00	15%	20,163
Navajo	107,449	16,309	535	12	0.00	0.033	30.09	20%	21,490
**New Mexico**									
McKinley	71,492	12,276	467	5	0.3	0.037	30.77	35%	25,022
Bernalillo	662,564	57,638	937	138	6	0.017	27.00	3%	19,877
Socorro	17,866	1,313	56	0	0.1	0.045	45.62	6%	1,072
Sandoval	131,561	11,962	231	0	1	0.019	33.80	1%	1,316
Cibola	27,213	3,521	112	4	0.3	0.031	35.14	9%	2,449
Rio Arriba	40,246	3,604	76	8	0.2	0.02	38.18	1%	402
San Juan	130,044	14,973	470	10	0.4	0.033	29.99	19%	24,708
**Utah**									
San Juan	14,746	1,903	37	1	0.00	0.02	37.73	21%	3,097
**Total**	1,409,120	152,722	3,680	219	-	-	32.30	-	163,222

**Fig 2:**
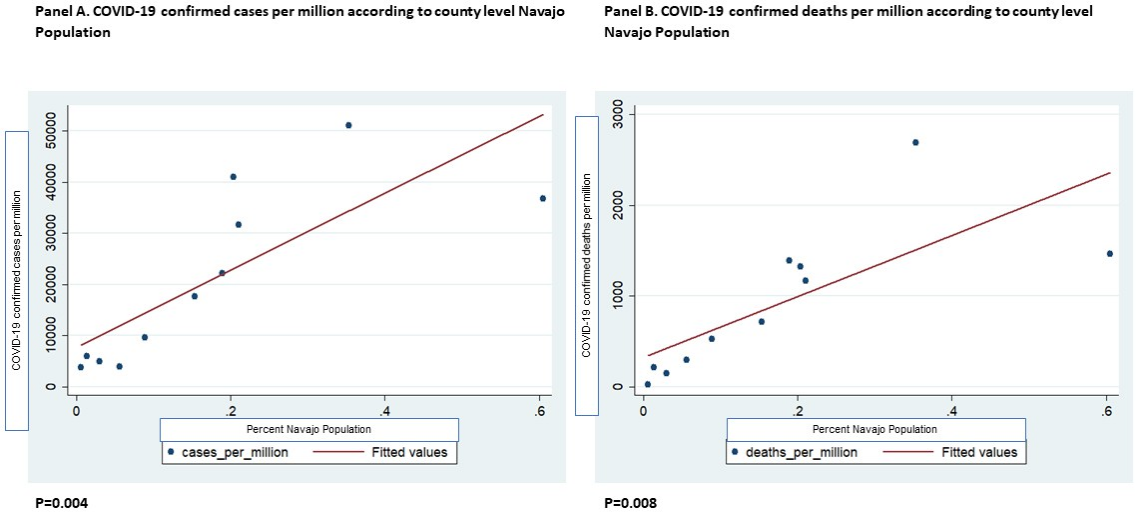
COVID-19 confirmed cases per million (A), deaths per million (B) according to county level Navajo Population (n = 11 counties represented in the Navajo Nation), July 18, 2020. Linear regression used, alpha is 0.05.

Utilizing linear regression with county as the unit, the percent Navajo population per county was a strong predictor of COVID-19 cases per million population (β = 138956.7, P = 0.004) and COVID-19 deaths per million population (β = 7272.19, P = 0.005) (Fig 2). The percent Navajo population per county did not have a statistically significant association with higher death-to-case ratio (β = .0245846, P = 0.697). Further, to explore whether health care access affects COVID-19 deaths, we added the total ICU beds per county and the estimated ICU beds needed to the regression model with COVID-19 deaths as the outcome. The percent Navajo population per county remained a strong predictor of COVID-19 deaths per million(β = 6607.434, P = 0.010), after adjustment for total ICU bed availability and the estimated ICU beds needed.

**Fig 3:**
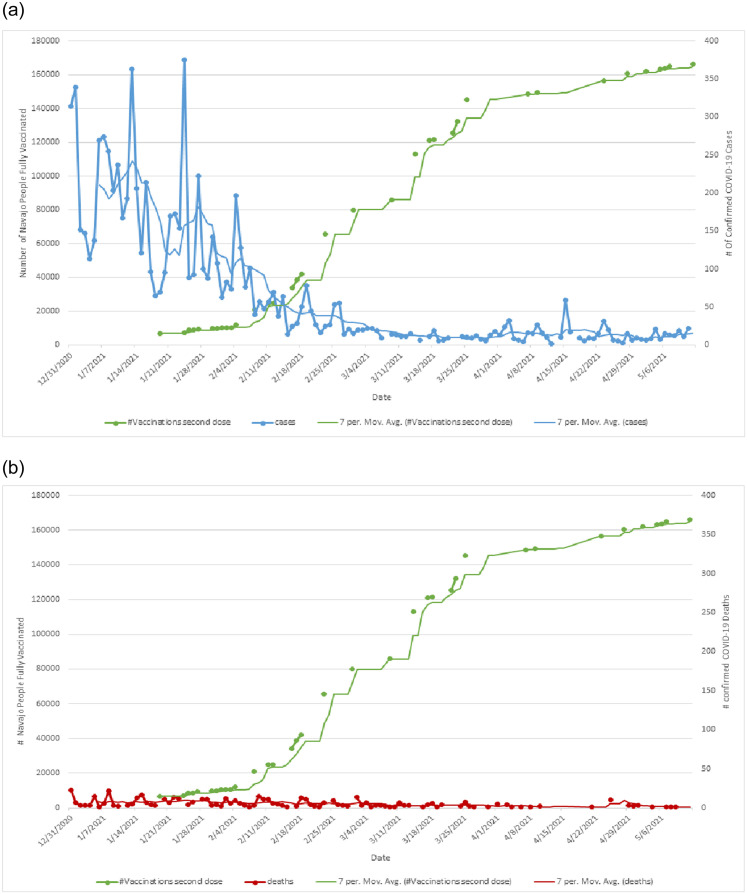
COVID-19 confirmed cases, COVID-19 confirmed deaths and vaccinations on the Navajo Nation. A. Number of confirmed COVID-19 cases, by county over 1 year on the Navajo Nation. B. Number of confirmed COVID-19 deaths, by county over 1 year on the Navajo Nation.

Lastly, an increase in the vaccinated Navajo Nation population from January-May 2021 is inversely associated with a precipitous drop in COVID-19 cases and deaths (Fig 3).

## Discussion

Early in the pandemic, the SARS-CoV2 virus landed in the middle of the Navajo reservation where the closest emergency room was likely to be 50 or more miles away. Many patients were transferred as much as 100 miles to the nearest medical center hospital on the Navajo Nation to receive further health care [22]. Overall, there was a substantial increase in the number of COVID-19 cases with a doubling time of 10.12 days on Navajo Nation early in the pandemic (from March 16, 2020- July 16, 2020) elongating to 32.3 days one year later by March 2021. Early in the pandemic, the percent Navajo population was a strong predictor of COVID-19 cases and deaths per million. Later in the pandemic, COVID-19 vaccinations were inversely associated with COVID-19 cases and deaths in the Navajo Nation. These findings are consistent with prior studies suggesting race/ethnic minority groups have higher risk of COVID-19, for example studies in African Americans and Latine populations in the US [8,9,23]. Counties with a higher percentage of Black residents had higher cases and deaths of COVID-19 [24]. Furthermore, predominantly Black communities accounted for 52% of COVID-19 diagnoses and 58% of COVID-19 deaths, nationally [24]. These disparities in COVID-19 outcomes may be attributed to inequitable distribution of social determinants of health. A related set of social determinants of health likely underpin our findings in the Navajo Nation.

### Susceptibility

A recent commentary highlights why Indian tribes may have been more susceptible to COVID-19 infection and death [25]. With most states advising residents to stay home to social distance and wash hands frequently to avoid coronavirus infection, approximately 70,000 Navajo Nation residents sheltered-in-place in homes without electricity and running water as only 33% of residents have access to clean running water on the Navajo Nation. The virus spread in towns that have no central utilities [22]. Lack of running water has also been shown to contribute to increased COVID-19 in American Indian tribes in other parts of the US, like Oklahoma [11]. Additionally, Navajo families live in close proximity to one another, and often live in multigenerational households which may accelerate coronavirus infections. Much of the Navajo Nation has no internet service, so families at home are unable to access timely public health information and may not able to schedule virtual visits with health care workers. Transportation to local townships with health care facilities is difficult due to few major roads on the Navajo Nation, most are unpaved, and little public transportation. These structural inequities are barriers to access food, water, sanitary supplies, and health care services. Several reports document environmental health hazards which compound these basic structural inequities [26–28]. These factors are compounded by a 44% poverty rate and high unemployment; both are large problems facing the Navajo people [7].

### Vaccine effectiveness

This manuscript highlights an association between percent Navajo population within counties in the Navajo Nation and the cases and deaths attributed to COVID-19 at the height of the pandemic. It also shows the acute downturn of cases and deaths after vaccination was swiftly implemented. Vaccination campaigns on Navajo Nation were effective, with reports in May 2021 suggesting over 85% of people over 16 years of age vaccinated on the Navajo Nation.

### Limitations

The primary limitation of this epidemiological analysis is that individual level data could not be used to understand the true impact of COVID-19 on the Navajo Nation. Using data at the county level and not individual data may result in ecological fallacy, this is a potential shortcoming of this work. Thus, the data viewed here should be considered preliminary. In addition, the percent of Navajo population in counties were available only from 2010, a more recent enumeration would be beneficial. Our team has approached the Navajo Nation Epidemiology Center to consider a collaborate upon future studies approved and co-led by Navajo Nation epidemiologists to explore confounding factors by analyzing individual data: age, sex, and comorbid conditions, and social determinants of health.

### Historical perspective

Recent news that COVID-19 cases and deaths are now low in the Navajo Nation, one may think that the time to be vigilant is over. However, the CDC warns of future threats to American Indians from emerging infectious diseases. Looking back, the story is not new. In 1993, the Navajo Nation faced another viral infectious disease, the Sinombre hantavirus, the etiologic agent in a highly fatal respiratory disease. At that time hantavirus caused an outbreak that was exacerbated by lacking infrastructure and poor public health conditions, very similar to the COVID-19 pandemic today [29].

### Navajo Resilience

In this manuscript we would like to highlight a positive story, showcasing the resilience of the Navajo people themselves, who have proven resilient through historical life challenges. For example, the Navajo Nation Community Health Representatives program [30] relays culturally-responsive, trusted care to traditional Navajo people and this program has been effective in vaccinating and educating Navajo people about COVID-19 in rural settings to prevent resurgence, transmission, and death from the virus. The Navajo Nation Department of Health, Navajo Area Indian Health Service (IHS) and state public health departments have coordinated their efforts with local Navajo communities on vaccination against COVID-19 across the vast and rural Navajo Nation. Their efforts have achieved a high percentage of fully vaccinated Navajo people. Furthermore, Navajo Nation President Jonathan Nez referenced the SARS-CoV2 virus as a monster, not unlike the monsters the Navajo overcame in traditional teachings. The emphasis to ‘slay the monster’ put on members of the tribe by carrying a ‘protective shield’ conferred by vaccination, to safeguard themselves and members of the tribe. With this message of vaccination and cultural inclusion, the people on the Navajo Nation have emerged through their own resilience and strength in combating COVID-19.

### Implications for health policy

The time to act upon structural differences is now [30]. Without basic infrastructure to appropriately respond to infectious diseases, like COVID-19 and its variants, the Navajo Nation will risk a resurgence or spread of new infectious diseases. From a health policy perspective, a greater implementation of strong health policies must also parallel true health needs, as defined by the Navajo people themselves. In honoring treaty responsibilities, the US federal government should provide greater sustained federal funding to Indian tribes to help them meet their infrastructure needs and challenges today. Similarly, tribal leadership, guidance and partnership is required to develop long-term solutions that honor the wishes of the Navajo people and also address basic infrastructure and health care necessities on the Navajo Nation. The combination of Navajo resilience and increased funding levels could be used to create and run hospitals, rural clinics, and public health programs to stop a resurgence of COVID-19 and new public health emergencies on the Navajo Nation.
